# Monosynaptic Stretch Reflex Fails to Explain the Initial Postural Response to Sudden Lateral Perturbations

**DOI:** 10.3389/fnhum.2017.00296

**Published:** 2017-06-07

**Authors:** Andreas Mühlbeier, Christian Puta, Kim J. Boström, Heiko Wagner

**Affiliations:** ^1^Department of Movement Science, University of MünsterMünster, Germany; ^2^Center for Interdisciplinary Prevention of Diseases Related to Professional Activities, Friedrich Schiller University JenaJena, Germany; ^3^Department of Sports Medicine and Health Promotion, Friedrich Schiller University JenaJena, Germany

**Keywords:** postural reflex, sudden perturbations, monosynaptic stretch reflex, polysynaptic reflex, spinal stability, chronic low back pain

## Abstract

Postural reflexes are essential for locomotion and postural stability, and may play an important role in the etiology of chronic back pain. It has recently been theoretically predicted, and with the help of unilateral perturbations of the trunk experimentally confirmed that the sensorimotor control must lower the reflex amplitude for increasing reflex delays to maintain spinal stability. The underlying neuromuscular mechanism for the compensation of postural perturbations, however, is not yet fully understood. In this study, we applied unilateral and bilateral sudden external perturbations to the trunk of healthy subjects and measured the muscular activity and the movement onset of the trunk. We found that the onset of the trunk muscle activity is prior to, or coincident with, the onset of the trunk movement. Additionally, the results of our experiments imply that the muscular response mechanism integrates distant sensory information from both sides of the body. These findings rule out a simple monosynaptic stretch reflex in favor of a more complex polysynaptic postural reflex mechanism to compensate postural perturbations. Moreover, the previously predicted negative correlation between reflex delay and reflex gain was also confirmed for bilateral perturbations.

## Introduction

People suffering from chronic low back pain (CLBP) exhibit delayed postural reflexes (Magnusson et al., 1996; Hodges and Richardson, 1998; Radebold et al., 2000, 2001; Leinonen et al., 2001; Reeves et al., 2005; Abboud et al., 2017) which are supposed to have an influence on the stability of the human spine. The physiological origin of the delay is still not fully understood. Hence, further insight into the existing mechanisms in healthy individuals is important as it may help to understand the pathophysiology of CLBP patients who may rely on different neuromuscular strategies following sudden perturbations.

Franklin and Granata (2007) and Liebetrau et al. (2013) performed model-based spine stability analyses and found a negative correlation between reflex delay and reflex amplitude. They predicted that a delayed muscular response requires its amplitude to be decreased in order to not destabilize the spine. This model prediction has been experimentally confirmed by applying sudden unilateral perturbations to the trunk (Liebetrau et al., 2013). Lateral perturbations induce a specific reaction pattern of the postural regulation where the contralateral trunk muscle response is significantly faster and has a higher reflex amplitude compared to the ipsilateral muscular response (Wulf et al., 2012; Cort et al., 2013; Liebetrau et al., 2013) (See the Materials and Methods Section for a precise definition of ipsilateral and contralateral stimulus condition). To our knowledge, there are no results concerning trunk muscle responses following perturbations that load both the left and right body side simultaneously. Mechanically, the simultaneity and symmetry of bilateral perturbations prevent the production of a moment of force on the trunk with the result that the trunk remains in an equilibrium in the frontal plane. It is, therefore, interesting to investigate the relation between reflex delay and reflex amplitude under such circumstances. There is actually no need for the central nervous system (CNS) to initiate a fast muscular response, so one would expect only little trunk muscle activity as compared to unilateral perturbations. Additionally, the neuromuscular mechanism implemented in the computational models of Franklin and Granata (2007) and Liebetrau et al. (2013) was just a simple monosynaptic stretch reflex. Hence, when the trunk is not deflected and keeps its equilibrium during bilateral perturbations, there is little to no stretch of the trunk musculature, and thus any occurrence of considerable trunk muscle responses would speak in favor of a more complex, polysynaptic reflex mechanism.

Another interesting aspect arises from a less mechanical and more physiological point of view. Hodges et al. (2001) and Leinonen et al. (2002) examined the temporal relation between the kinematics and the muscular response onset following trunk perturbations. They reported paraspinal reflex responses occurring simultaneously or even prior to the movement of the trunk^1^. It is virtually impossible for a monosynaptic stretch reflex in the trunk musculature to trigger such a fast response to a postural perturbation. Hodges et al. (2001) consequently presumed that the neuromuscular mechanism initiating the response must receive its input from more distant parts of the body, e.g., from muscle and/or joint receptors in the perturbed upper limb.

In the present study, we tested this assumption by using a perturbation paradigm that allowed to trigger distant mechanisms while excluding an initiation by local mechanisms. Following Hodges et al. (2001), we attribute the term “local” to those response mechanisms whose afferent input is located in the responding muscle itself or in direct proximity to it (here, type Ia, Ib, or II afferents in the muscle spindles, Golgi tendon organs, etc., of the trunk musculature), while “distant” is defined as non-local and refers to all remaining possible mechanisms (here, tactile receptors in the hand, muscle, and/or joint receptors in the arm, elbow, shoulder, etc.). Equivalently, the terms “local” and “distant” can also apply to receptors and then refer to those receptors that are located within the responding muscle itself or distant to it, respectively. Throughout this paper, we consider responses of the trunk musculature only, hence “local” refers to mechanisms and receptors that are located within the responding trunk muscle.

**Figure 1:**
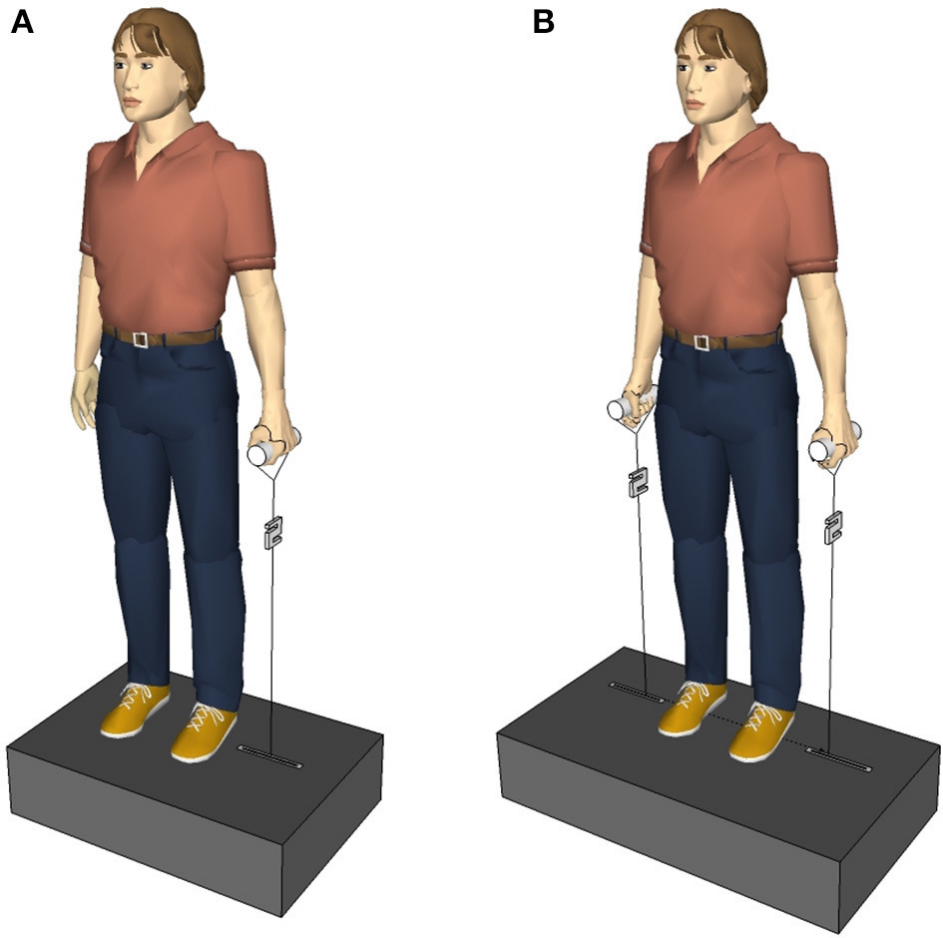
**(A)** First experimental setup. The subjects held a single handle with the left and right hand, one after the other. **(B)** Second experimental setup. The subjects held a handle in each hand. The perturbations randomly occurred via the left, the right, or both handles.

The trunk was perturbed laterally and indirectly via vertically pulled handles that the subjects held in their hands. The experimental setup (Figure 1) allowed the application of one-handed (unilateral) and two-handed (bilateral) perturbations. A unilateral perturbation stretched the upper limb grasping the handle, and deflected the subject's trunk. Thus, it permitted both the initiation of distant mechanisms, e.g., by receptors in the upper limb, and the initiation of local mechanisms, e.g., by receptors in the trunk muscles contralateral to the side of the perturbation. Conversely, a bilateral perturbation would have a symmetrical effect to the subject's posture without deflecting the trunk and stretching trunk muscles, so any considerable response of the trunk musculature would imply the participation of distant mechanisms.

Since contralateral trunk muscle responses occurred significantly earlier than ipsilateral responses (Wulf et al., 2012; Cort et al., 2013; Liebetrau et al., 2013), and since Hodges et al. (2001) and Leinonen et al. (2002) showed that the first trunk muscle response to an external perturbation occurred earlier than the corresponding trunk movement, a distant mechanism is likely to initiate the contralateral trunk muscle response.

For the bilateral stimulus, the upper limbs on both sides of the body are stretched, and hence there are two possibilities for a distant reflex mechanism to work: Either only receptors in the contralateral (antagonistic) side of the body trigger the mechanism, or receptors in both sides. In the first case, the response to the bilateral stimulus should not be different from the response to the contralateral stimulus, while in the second case there can be, but does not need to be, a difference. Due to a more effective antagonistic functioning of skeletal muscles, the first case appears to be more plausible.

The above physiological considerations lead to the following assumptions:

A1: If an exclusively local mechanism initiates the muscular activity, the kinematic response should always be prior to the muscular response.
A2: If an exclusively local mechanism initiates the muscular activity, there should be no significant muscular response to both the ipsilateral and bilateral stimulus.
A3: If a distant mechanism purely relying on sensory information from the contralateral side of the body initiates the muscular activity, the response to the bilateral stimulus should not differ from that caused by the contralateral stimulus.

In line with Hodges et al. (2001) and Leinonen et al. (2002), we expect a distant mechanism to trigger the trunk muscle activity. Additionally assuming that only contralateral (antagonistic) receptors contribute to the distant mechanism, using the above assumptions A1–A3 we arrive at the following three hypotheses:

H1: The kinematic response occurs later than, or coincident with, the muscular response (So that according to assumption A1 an exclusively local mechanism can be excluded).
H2: There is a significant muscular response following a bilateral perturbation (So that according to assumption A2 an exclusively local mechanism can be excluded).
H3: The muscular response following a bilateral perturbation does not differ from the muscular response following the contralateral stimulus (So that according to assumption A3 a distant mechanism solely relying on the contralateral receptor information is responsible for the initial muscular response).

In addition to that, we aim to generalize the model-based predictions of Franklin and Granata (2007) and Liebetrau et al. (2013) by hypothesizing that

H4: The muscular responses following contralateral, ipsilateral, and bilateral stimuli exhibit a negative correlation between reflex delay and reflex amplitude.

To test these hypotheses, two different experiments were conducted. In the first experiment, we applied unilateral perturbations and used surface electromyography (sEMG) and motion analyses to determine the temporal order of kinematic response onset and reflex response onset. In the second experiment, we compared trunk muscle responses following unilateral and bilateral perturbations with respect to reflex delay and reflex amplitude.

## Materials and methods

### First experiment: temporal order of kinematic and reflexive onsets

#### Experimental procedure

##### Subjects

Thirteen healthy female subjects [mean ± standard deviation (*SD*); age: 31 ± 9 years, body mass: 59 ± 8 kg, and body height: 168 ± 7 cm] participated in the study after they signed their informed consent form that had been approved by the local ethics committee of the University of Jena and conformed to the Declaration of Helsinki. None of the tested persons suffered from chronic back pain.

##### Experimental setup and procedure

The subjects stood relaxed, looking straight ahead, and holding a handle with their elbows extended in the right or left hand, respectively (Figure 1A). The arm, the hand, and the lateral malleolus formed an imaginary line. The handle was connected to a servo motor (Stromag Elektronik GmbH, FLP 31/0125–30AA232) via an inelastic, high-modular, kevlar-cored, aramid string (Edelrid, Germany). A load cell (50–2,000 N, 2 kHz, Biovision, Wehrheim, Germany) was inserted into the string between the handle and the servo motor to measure the force applied to the hand. In total, 10 lateral perturbations were applied to each subject; first 5 via the left hand, then 5 via the right hand. Considering the force (~150 N) and duration (100 ms) of the perturbation, the loading was quite abrupt and sudden causing a slight deflection of the subject's trunk in the frontal plane. Between two perturbations, if necessary, a correction of the subject's position was given. The contralateral and ipsilateral muscles were defined referring to the side of perturbation. Thus, when the force was applied to the left handle, the left trunk muscles were defined as ipsilateral and the right trunk muscles were defined as contralateral, and vice versa for the right perturbation. Equivalently, the terms “ipsilateral stimulus” and “contralateral stimulus” refer to the perturbation being applied to the same and opposite side of the measured muscle, respectively.

##### Electromyography

An sEMG (5–700 Hz, Biovision, Wehrheim, Germany) of five trunk muscles [*M. obliquus externus abdominis* (OE), *M. obliquus internus abdominis* (OI), *M. rectus abdominis* (RA), *M. multifidus pars lumborum* (MF), *M. erector spinae pars lumborum* (ES)] was recorded using pairs of disk electrodes (H93 Arbo, Ag/Ag-Cl Sensor, Tyco Healthcare, Neustadt, Germany; diameter 0.5 cm, distance 2.5 cm). The electrode placement was done according to internationally established recommendations (Ng et al., 1998; Hermens et al., 1999). The reference electrodes of the bipolar montage (abdominal trunk muscles) were positioned bilaterally on the tibia. The monopolar reference electrodes (paraspinal muscles) were placed at the thoracolumbar spinous processes at the level of the 12th thoracic vertebra. The skin was shaved and cleaned with medical abrasive paste (EPICONT, Marquette Hellige GmbH, Freiburg, Germany). The raw sEMG data were collected at a sampling rate of 2 kHz (AD-transformation, DAQCard-AI-16E-4: 12 bit, National Instruments, USA) and preamplified 2,500 times (bipolar) and 5,000 times (monopolar), respectively.

##### Kinematic of the trunk and spine

Three-dimensional kinematic data of the subject's movements of the trunk and spine were collected using an infrared based motion capture system (Qualisys, Sweden). Four cameras were positioned 1.5–2 m behind the subjects and calibrated using a calibration-frame with four fixed markers. Five infrared-reflective markers were attached to the skin at the contralateral shoulder (CLSH), the ipsilateral shoulder (ILSH), the 7th cervical vertebra (C_7_), the 1st and 5th lumbar vertebra (L_1_ and L_5_) to measure the kinematic onset of different body segments. The palpatory localization of the bony landmarks was done by two experienced investigators. Position data were sampled at 120 Hz and automatically converted into three-dimensional co-ordinates. The kinematic and sEMG measurements were started synchronously via a switch.

#### Data analysis

The processing of the force and sEMG data was performed using MatLab (MathWorks, USA).

##### Force

The onset of the perturbation was identified via the force impact using an automatic algorithm and was defined as the time when the force reached 20% of the maximum force above preloading baseline. The automatic detection and the following visual check were made for each perturbation onset (five left and five right perturbations per subject). The force signal was used as trigger signal to detect the latencies of the sEMG and kinematic responses.

##### sEMG and kinematic onsets

The sEMG of seven trunk muscles and the position data of five marker locations were analyzed. All sEMG signals were centered and high-pass filtered (4th-order Butterworth filter, 40 Hz) to avoid influences from movement artifacts. To remove high frequency noise a moving average filter (21 samples) was applied to the rectified sEMG signal. sEMG and kinematic latencies were defined as the time from beginning of force impact to the first onset of the reflex or motion response, respectively. Reflex and kinematic onsets were defined as the instant when the signal values exceeded 4 *SD* above the baseline activity (300 ms) within a time interval up to 200 ms following the force impact. This time constraint was imposed to exclude contributions of voluntary responses (Thomas et al., 1998; Granata et al., 2004). The medians of five left and five right muscle responses and kinematic responses, respectively, were used for further calculations.

#### Statistical analysis

The sEMG onset and the kinematic onset of the respective spinal segment were analyzed by a repeated-measures analysis of variance (ANOVA) using a 2 × 5 test design with the following within-subject factors: “spinal segment” (L_1_/L_5_) and “response latency” (latency of four corresponding trunk muscles/latency of the corresponding spinal marker) in order to reveal differences in the delay of the spinal marker and its corresponding trunk muscles (L_1_-marker compared to the contralateral and ipsilateral ES1 and ES2; L_5_-marker compared to the contralateral and ipsilateral MF1 and MF2).

All ANOVA results were corrected for violation of sphericity using the Greenhouse-Geisser approach for epsilon correction of degrees of freedom. Epsilon was given if appropriate. The Bonferroni method was used for all *post-hoc* multiple comparisons. All effects are reported as significant at *p* < 0.05. The calculations were performed using SPSS (IBM SPSS Statistics, USA).

### Second experiment: unilateral vs. bilateral reflex responses

#### Experimental procedure

##### Subjects

Twenty healthy subjects (mean ± *SD*; age: 21 ± 2 years, body mass: 66 ± 11 kg, and body height: 175 ± 9 cm) volunteered to participate in the second examination, 11 women and nine men. Each subject was informed precisely of the procedures in this study and gave his written consent. The tested persons neither suffered from chronic back pain nor did they participate in the first experiment.

##### Experimental setup and procedure

As the first and second experimental setup were similar, here after only the differences to the first experimental setup are mentioned. The subjects stood upright on a metal construction containing two servo motors (AKM 44 E, Kollmorgen, Germany; controlled by a Servo Drive S300, Danaher Motion, Germany) instead of one (Figure 1B). The perturbations occurred either on the right side or on the left side or on both sides at the same time. Three sets with 24 perturbations each were applied to one subject, 72 perturbations in total per subject. Within a single set the subject experienced eight right perturbations, eight left perturbations, and eight simultaneous perturbations on both sides occurring in a randomized order that varied from subject to subject. Between two perturbations of one set there was an inter-perturbation pause of 3 s so that subjects had enough time to get back in the upright position, if necessary, before the next perturbation occurred. Between two sets there was an inter-set pause of 3 min. The perturbation force was adapted to each subject individually as $F\left[N\right]=16\%\cdot 9.81\frac{m}{s^{2}}\cdot M\left[kg\right]$, with the subject's body mass M, e.g., a subject of M = 65 kg was exposed to perturbations with a force of F = 102 N.

##### Electromyography

The sEMG of the lumbar ES, the lumbar MF, and the OE was recorded. The electrode placement was done according to Hermens et al. (1999) and Ng et al. (1998). The common ground electrode was placed at the 7th cervical vertebra. After the skin had been shaved and cleaned with medical abrasive paste (EPICONT, Marquette Hellige GmbH, Freiburg, Germany), disposable Ag-AgCl electrodes (H93SG Arbo, Tyco Healthcare, Neustadt, Germany) with a circular uptake area of 1 cm in diameter were placed with an inter-electrode distance of 2.5 cm. Muscle activity was measured bipolar at a sampling rate of 2 kHz (preamplified 2,500 times; Biovision, Germany). Each subject performed all three sets in a single session so that the electrodes were applied once and all instrumentation settings remained constant.

#### Data analysis

The processing of the force and sEMG data was performed using MatLab (MathWorks, USA).

##### Force

The onset of the perturbation was defined as the instant when the force signal reached 10% of the maximum force above the preloading baseline. The reflex onset was detected using the force signal.

##### sEMG

The sEMG signals were high-pass filtered (4th-order Butterworth filter, 40 Hz), rectified, and smoothed by ±10 samples moving average. The reflex onset was defined as the instant when the signal value exceeded 4 *SD* greater than the average of the preloading baseline activity (400 ms). The reflex amplitude was determined as the maximum value within the interval of 20–200 ms after response onset. Following a bilateral perturbation both left and right muscle responses were taken into account, while ipsilateral and contralateral were defined as in the first investigation. For every muscle and subject the medians of 48 ipsilateral (24 left and 24 right), 48 contralateral, and 48 bilateral muscle responses were taken into account for the statistical analysis.

#### Statistical analysis

The reflex delay data were analyzed by a repeated-measures ANOVA using a 3 × 3 test design with the within-subject factors “stimulus condition” (ipsilateral/contralateral/bilateral) and “muscle” (OE/ES/MF). The ANOVA was followed by Bonferroni-corrected, *post-hoc* multiple-comparison tests against stimulus condition. Concerning the data of the reflex amplitude, the same calculation procedure was used as for the reflex delay described above. All results of repeated-measures ANOVA were corrected for violation of sphericity using the Greenhouse-Geisser approach for epsilon correction of degrees of freedom. Epsilon was given if appropriate. All effects are reported as significant at *p* < 0.05. To assess the linear relationship between reflex delay and reflex amplitude following the ipsilateral, contralateral, and bilateral stimulus, Pearson's correlation coefficient *r* and the corresponding *p*-value were calculated. The *p*-values were statistically corrected using the Holm-Bonferroni method. We also calculated the coefficient of determination *R*^2^ giving the proportion of variance of the reflex amplitude that is determined by the variance of the reflex delay (For linear regression, *R*^2^ coincides with the square of Pearson's correlation coefficient, so *R*^2^ = *r*^2^). The statistical calculations were performed using SPSS (IBM SPSS Statistics, USA) and MatLab (Mathworks, USA).

## Results

### First experiment: the response onset is prior to, or coincident with, the kinematic onset

**Figure 2:**
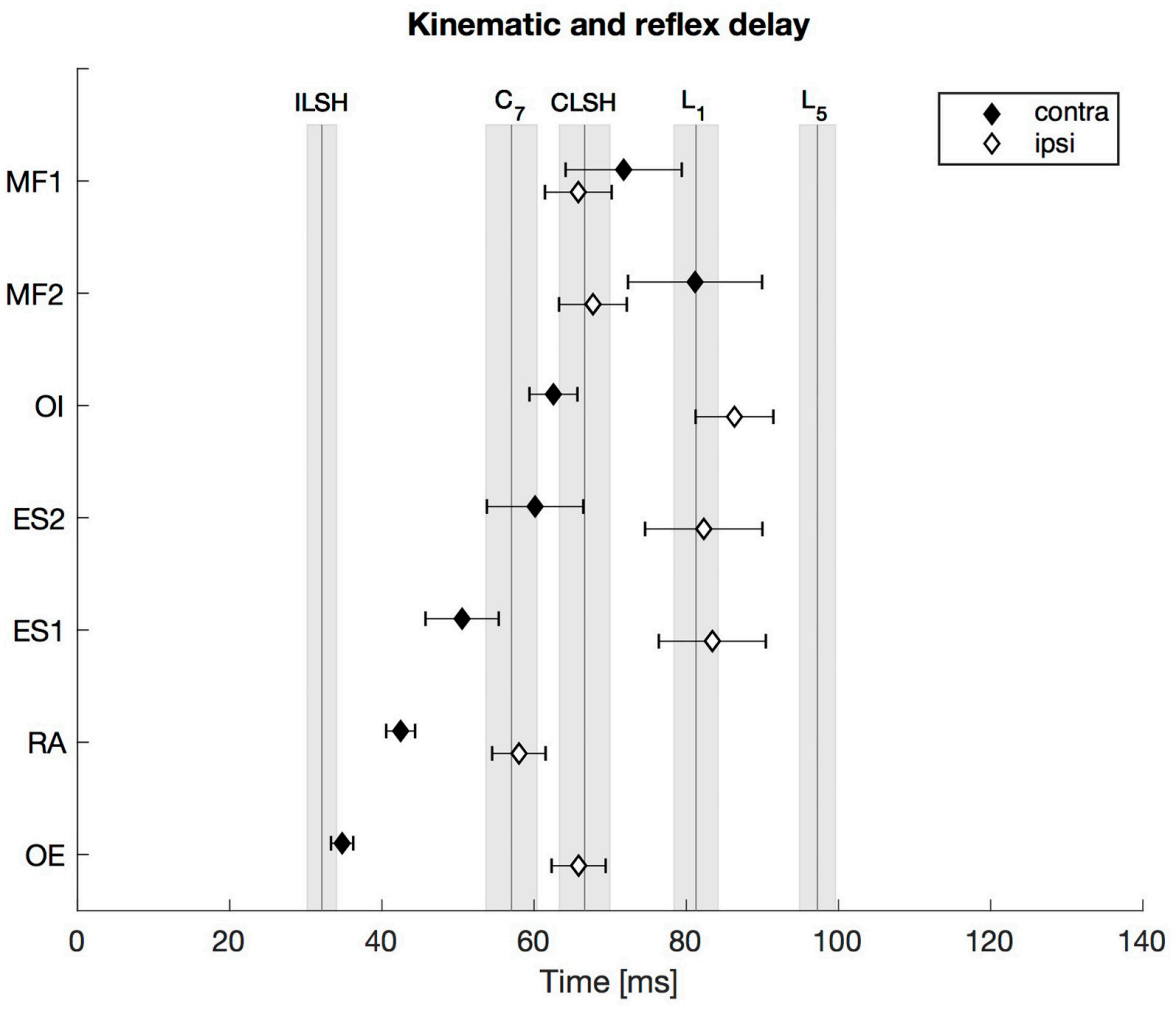
First experiment: Kinematic and reflex delay following the ipsilateral and the contralateral stimulus. The vertical lines indicate the mean onset delay of the five different markers (ILSH, ipsilateral shoulder; CLSH, contralateral shoulder; C_7_, 7th cervical vertebra; L_1_, 1st lumbar vertebra; L_5_, 5th lumbar vertebra), while the shaded surfaces refer to the respective standard error of the mean (SEM). The diamonds assign the contralateral (contra) and ipsilateral (ipsi) mean trunk muscle reflex delay with the SEM indicated by the respective error bars. Note that all trunk muscle responses occurred prior to, or not significantly later than, the movement onset of the two spinal segments L_1_ and L_5_. *N* = 13.

The upper body's response to the unilateral postural perturbation resulted in a typical response pattern as shown in Figure 2. After the onset of the perturbation at *t* = 0 ms the marker at the ipsilateral shoulder showed the first kinematic response after 32 ms, followed by the marker at C_7_ (57 ms) and the marker at the contralateral shoulder (66 ms). The markers along the lumbar spine were of particular interest since they are anatomically close to the location of the measured muscles. The response onsets of all muscles, contralateral as well as ipsilateral, were prior to the kinematic onset of the L_5_-marker (97 ms). Furthermore, the ipsilateral reflex delays were significantly longer than the contralateral ones (except MF muscles, see Supplementary Material for significance levels).

To compare the onset delay of the sEMG with the kinematic response onset, the anatomical proximity of the L_1_- and L_5_-markers to the location of the ES- and MF-electrodes was used. Thus, the markers' motion onset was compared with the muscles' activity onset (L_1_ to ES1/ES2, L_5_ to MF1/MF2). The repeated-measures ANOVA (2 spinal segments × 5 corresponding response latencies) revealed a significant main effect of the factor “response latency” [*F*_(4, 48)_ = 4.812, $\eta_{p}^{2}=0.286$, *p* = 0.012] and no significant main effect of the factor “spinal segment” [*F*_(1, 12)_ = 0.552, $\eta_{p}^{2}=0.044$, *p* = 0.472]. Furthermore, there was a significant interaction between the factors “spinal segment” and “response latency” [*F*_(4, 48)_ = 7.864, $\eta_{p}^{2}=0.396$, *p* < 0.0001]. Bonferroni-adjusted *post-hoc* comparisons (α = 0.05/4; *p* < 0.0125) revealed that the onset of the sEMG response of the contralateral ES muscles occurred ~20–30 ms earlier than the kinematic response onset of the L_1_-marker, which resulted in a significant difference for the ES1 (*p* < 0.01) and an almost significant difference for the ES2 (*p* = 0.021). The responses of the ipsilateral ES muscles and the L_1_-marker were coincident, i.e., not significantly different. The sEMG onsets of the ipsilateral MF1 and MF2 were ~30 ms earlier (*p* < 0.0001) than the kinematic onset of the L_5_-marker, whereas the response onsets of the contralateral MF1 and MF2 were ~15–25 ms earlier than the L_5_-onset, which did not result in significant differences (MF1: *p* = 0.11, MF2: *p* = 0.027).

### Second experiment: the bilateral reflex delay is greater than the contralateral reflex delay

**Figure 3:**
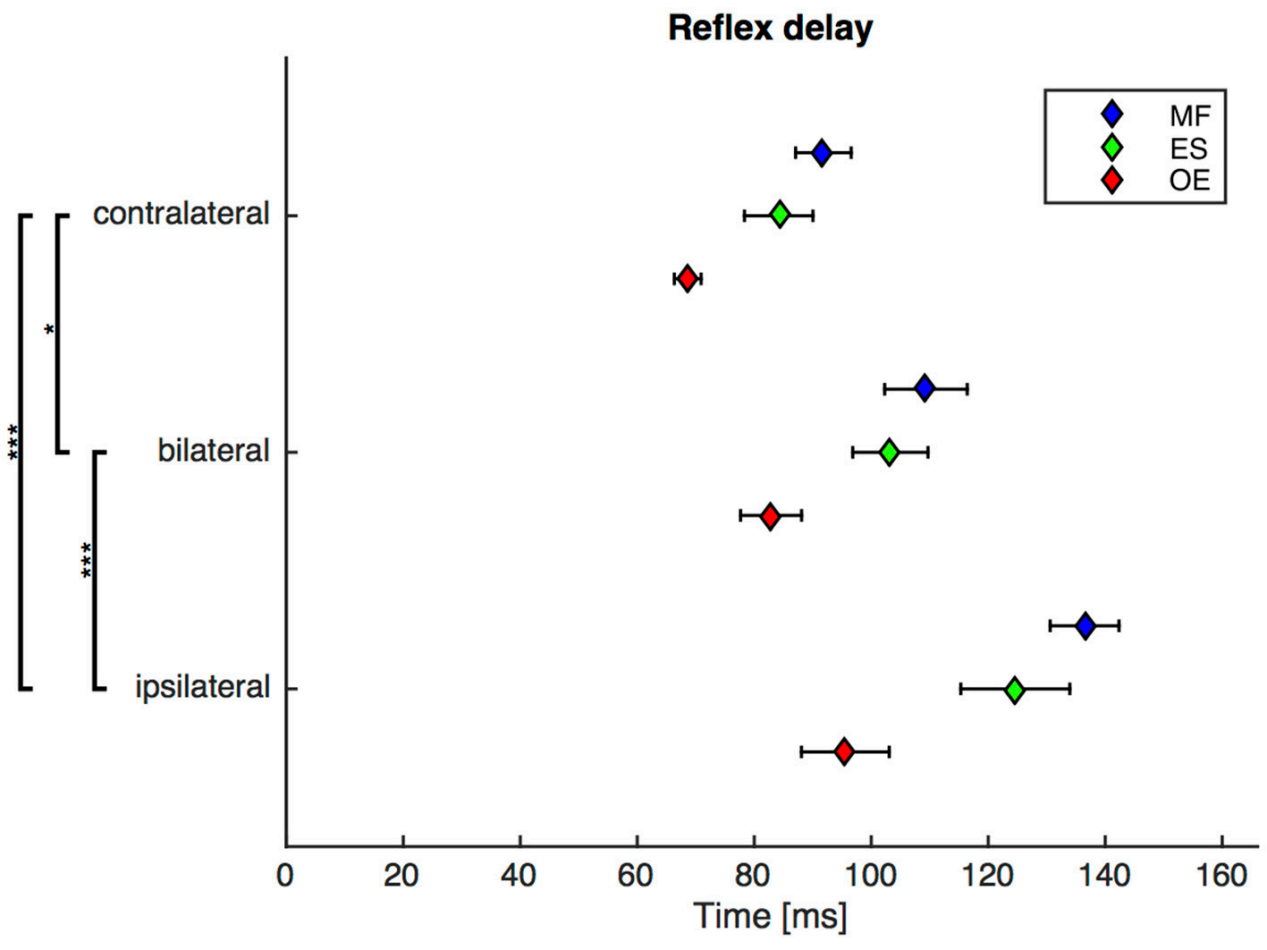
Second experiment: Mean and SEM of the reflex delay of *M. obliquus externus* (OE), *M. erector spinae* (ES), and *M. multifidus* (MF) following contralateral, bilateral, and ipsilateral stimuli. Brackets indicate significant differences in the reflex delay between the stimuli. *N* = 20.

A repeated-measures ANOVA revealed a highly significant main effect of the stimulus condition on the reflex delay [*F*_(1.6, 29.5)_ = 21.4, ϵ = 0.77, *p* < 0.001). *Post-hoc* pairwise comparisons showed that the reflex delay following the bilateral stimulus was significantly greater than the reflex delay following the contralateral stimulus [bilateral (mean ± SEM): 99 ± 5 ms, contralateral: 82 ± 3 ms; *p* < 0.05] and significantly shorter than the reflex delay following the ipsilateral stimulus [ipsilateral (mean ± SEM): 119 ± 6 ms; *p* < 0.001, Figure 3].

### Second experiment: the bilateral reflex amplitude is smaller than the contralateral reflex amplitude

**Figure 4:**
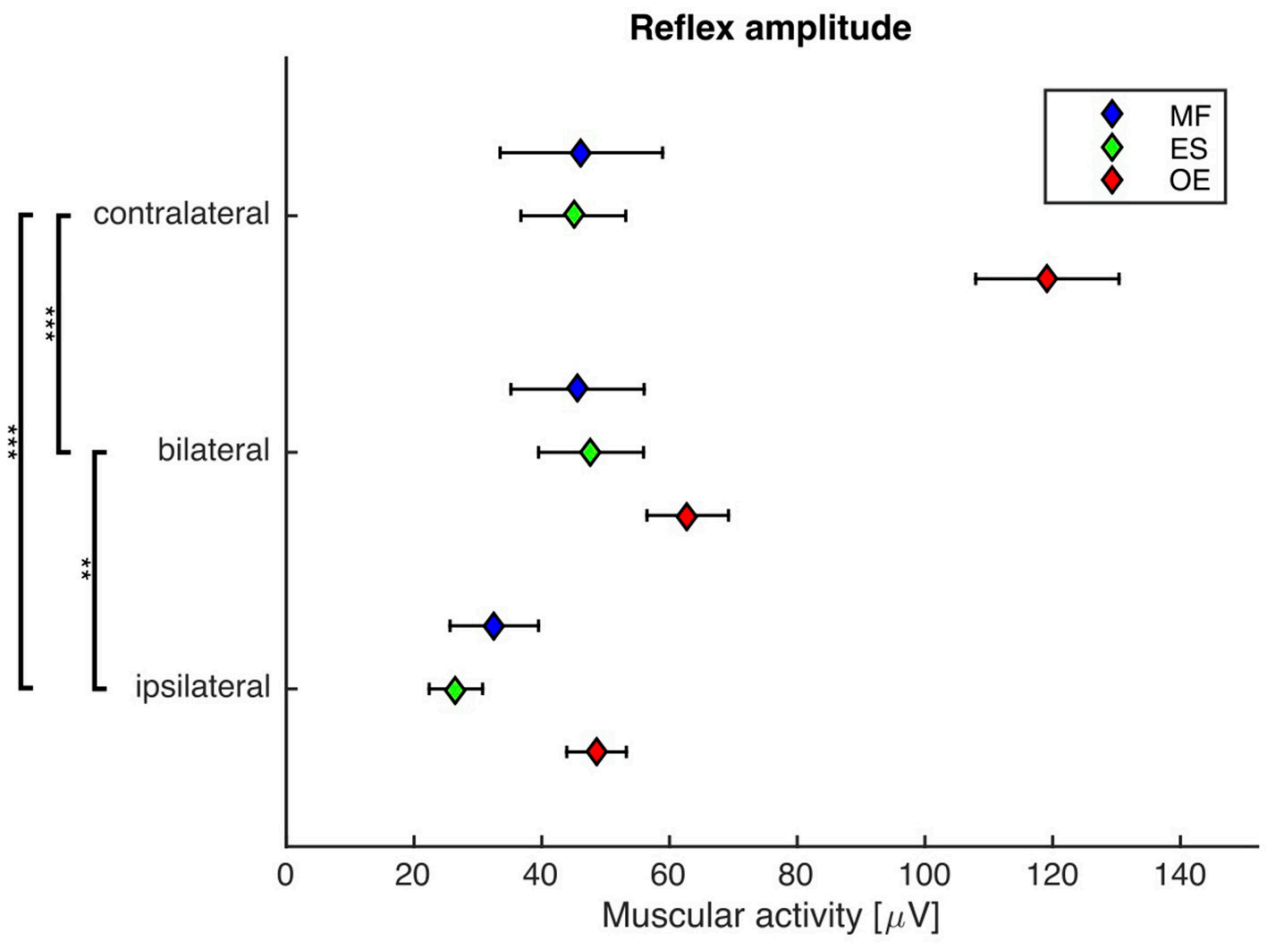
Second experiment: Mean and SEM of the reflex amplitude of *M. obliquus externus* (OE), *M. erector spinae* (ES), and *M. multifidus* (MF) following contralateral, bilateral, and ipsilateral stimuli. Brackets indicate significant differences in the reflex amplitude between the stimuli. *N* = 20.

A repeated-measures ANOVA revealed a highly significant main effect of the stimulus condition on the reflex amplitude (*F*_(2, 38)_ = 32.2, *p* < 0.001). *Post-hoc* pairwise comparisons showed that the reflex amplitude following the bilateral stimulus was significantly smaller than the reflex amplitude following the contralateral stimulus (bilateral: 52 ± 7 μV, contralateral: 70 ± 7 μV; *p* < 0.001), and significantly higher than the reflex amplitude following the ipsilateral stimulus (ipsilateral: 36 ± 4 μV; *p* < 0.01, Figure 4).

### Second experiment: the reflex delay negatively correlates with the reflex amplitude on all stimulus conditions

**Figure 5:**
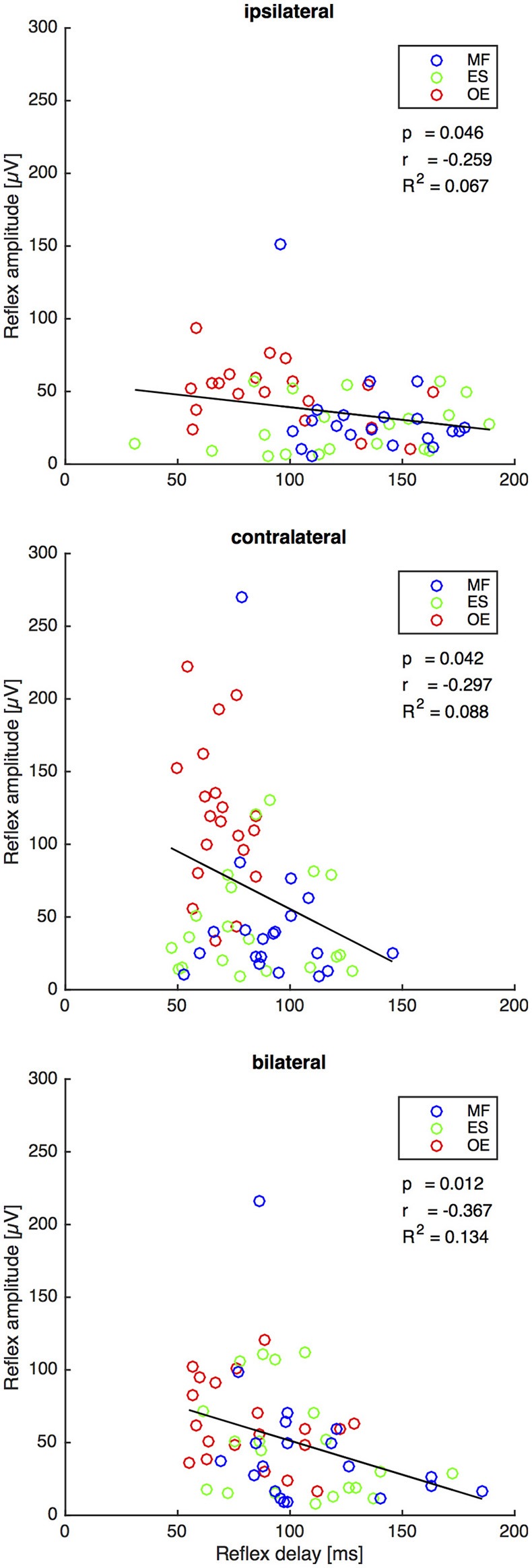
Second experiment: Reflex delay and reflex amplitude of the trunk muscles *M*. *obliquus externus* (OE), *M. erector spinae* (ES), and *M. multifidus* (MF) following the ipsilateral, the contralateral, and the bilateral stimulus. The bold lines indicate the linear regression of each pair of variables, whose slope equals Pearson's correlation coefficient *r* shown under the legend insets. The corresponding statistically corrected *p*-values *p* and the coefficients of determination *R*^2^ are also shown. *N* = 20.

For all stimuli there was a weak but significant anticorrelation between reflex delay and reflex amplitude (ipsilateral: *r* = −0.259, *p* = 0.046, *R*^2^ = 0.067; contralateral: *r* = −0.297, *p* = 0.042, *R*^2^ = 0.088; bilateral: *r* = −0.367, *p* = 0.012, *R*^2^ = 0.134, Figure 5).

## Discussion

The goal of this study was to get further insights into the neuromuscular mechanisms that initiate compensatory muscular responses to sudden perturbations. As the exact identification of the neuromuscular mechanism is very difficult due to the complexity of the postural regulation, the afferent sources were roughly categorized into local and distant receptors. On the basis of this rough categorization, conclusions about the participation of local neuromuscular mechanisms, such as the monosynaptic stretch reflex, can be drawn from the experimental results.

As for our first hypothesis, the kinematic response turned out to occur too late to be responsible for the muscular response. The condition for the occurrence of a monosynaptic stretch reflex is the lengthening of the muscle, which requires movement. As the muscular response was prior to, or coincident with, the movement of the respective markers in the unilateral condition (Figure 2), and as there was no lengthening of trunk muscles in the bilateral condition, the initiation of the muscular response by a monosynaptic stretch reflex or another exclusively local neuromuscular mechanism can be excluded. This finding is in concordance with the results of Hodges et al. (2001) and Leinonen et al. (2002) and indicates that there are trunk muscle responses which are not triggered by trunk movement.

As for our second hypothesis, our results show a considerable muscular response to the bilateral stimulus. Hence, once more an exclusively local reflex mechanism can be excluded.

The exclusion of a local mechanism implies the existence of a distant neuromuscular mechanism that underlies the trunk muscle activity. Against our third hypothesis, however, there is a significant difference between the muscular responses to contralateral and bilateral stimuli (Figures 3, 4). Thus, the distant neuromuscular mechanism that primarily contributes to the initial response relies on sensory information from both sides of the body, and not only from the contralateral (antagonistic) side.

Taken together, the initial response measured in our experiments is primarily induced by a distant neuromuscular mechanism. This mechanism relies on distant sources of sensory information from both sides of the body, in particular from the upper limbs, which interact and influence each other. Although a contribution of the local monosynaptic reflex loop to the initial part of the muscular response can be excluded, it is implausible to assume that the monosynaptic reflex is completely suppressed. Instead, it is plausible to assume, and not in contradiction with our data, that local sources of sensory information may still contribute to the muscular response in the later response components.

Since, as we have just concluded, the muscular response cannot be initiated by a local monosynaptic stretch reflex, a more complex, polysynaptic reflex mechanism must be involved. Cort et al. (2013) examined muscular responses of subjects kneeling on a robotic platform that caused sudden dynamic unilateral perturbations to the trunk. They reported an inhibition pattern on the ipsilateral muscle side, and an activation pattern on the contralateral muscle side, within the time interval 25–150 ms following the perturbation onset. As the bilateral perturbation is identical to two simultaneous unilateral perturbations, and supposing that the neuromuscular system tries to activate contralateral trunk muscles and to inhibit ipsilateral trunk muscles, it is reasonable to assume an interaction of the left and right mechanisms on the bilateral stimulus condition. Consequently, this interaction, such as a reciprocal inhibition of the α-motor neurons at the spinal level, may result in a decreased reflex amplitude and an increased reflex delay as compared to the contralateral stimulus condition.

The bilateral stimulus induced muscular responses with a reflex delay and a reflex amplitude that fall between those induced by contralateral and ipsilateral stimuli. In line with the model-based predictions for spinal stability (Franklin and Granata, 2007; Liebetrau et al., 2013), our findings reveal a weak but significant negative correlation between reflex delay and reflex amplitude in the ipsilateral, contralateral, and bilateral trunk muscle response (Figure 5), confirming our fourth hypothesis. In view of the increased delay of reflex responses in CLBP patients (Magnusson et al., 1996; Hodges and Richardson, 1998; Radebold et al., 2000, 2001; Leinonen et al., 2001; Reeves et al., 2005; Abboud et al., 2017), future research needs to examine if such increased delay can also be found following bilateral perturbations. A muscular response amplitude that is not adjusted to the delay leads to spinal instability, which would be a possible explanation for the reported back pain.

Note that the significant differences between the reflex amplitudes of the contralateral group and the two other groups (Figure 4) is mainly due to the *M. obliquus externus* (OE), which apparently responds the strongest to the applied perturbations. Since all muscles were treated the same way during the EMG procedure, a methodical reason for this phenomenon is unlikely. A more plausible explanation may be the following: Due to the lateral direction of the trunk perturbation applied in our experiments, the OE is better suited than the other measured muscles to compensate this type of perturbation. Other studies, where the trunk was perturbed in the anterior-posterior direction, show a higher participation of the ES and MF muscles and a comparably lower activation of the OE.

## Limitations of the study

Although the first and second experiments were almost identical, the sEMG results revealed some considerable differences. It turned out that the unilateral reflex delays of the EO, ES, and MF in the first experiment were about 30 ms shorter than in the second experiment. This difference may be explained first by different durations of the servo motors to produce the maximum force (time to rise to the maximum force amplitude: ~90 ms in the first experiment, ~20 ms in the second experiment). Thus, the onset of the perturbation in the second experiment was set earlier than in the first experiment, leading to an increased reflex delay. Second, the thresholds of the force signal were determined in a different manner (first experiment: 20% of the maximum force, second experiment: 10%). Third, in the first experiment, the perturbation force of the servo motor was always the same for all subjects (force: 150 N, body mass: 57 ± 5 kg), whereas in the second experiment the force was adjusted individually to the body mass of each subject (force: 104 ± 17 N; body mass: 66 ± 11 kg) to prevent injuries in lightweight subjects. Fourth, there may have been a higher muscle pre-activation in the first experiment, because the subjects, holding only one handle, knew which handle would be pulled, and so the CNS may have adapted the muscular response to the expected perturbation. However, a re-test of the first experiment using the setup of the second experiment and a small group of subjects did not lead to a shorter reflex delay.

## Conclusions

Our results provide evidence that the initial muscular response to sudden postural perturbations is not caused by a local monosynaptic stretch reflex of the trunk musculature. Instead, there must exist a more complex, polysynaptic neuromuscular reflex mechanism using distant sensory information from both sides of the body. Furthermore, our findings reveal a negative correlation between reflex delay and reflex amplitude, which confirms the model-based predictions of Franklin and Granata (2007) and Liebetrau et al. (2013).

## Ethics statement

The study was carried out in accordance with the Declaration of Helsinki and was approved by the ethics committee of the Friedrich Schiller University Jena (3039-02/11 and 0558-11/00). All subjects gave written informed consent after having been thoroughly informed about the nature and course of the experiment.

## Author contributions

HW and CP conceived the project and ordered the construction of the apparatus. CP was responsible for the data acquisition and conduction of the first experiment, whereas AM conducted and acquired the data of the second experiment. HW, CP, KB, and AM analyzed and interpreted the data. All authors worked on the manuscript, approved the final version to be published, and are accountable for all aspects of the work.

### Conflict of interest statement

The authors declare that the research was conducted in the absence of any commercial or financial relationships that could be construed as a potential conflict of interest.

## Footnotes

^1^Hodges et al. (2001) detected a postural response of the *M. erector spinae* and the *M. transversus abdominis* (both ~50 ms) that was almost coincident with the movement of the trunk (S_1_, ~40 ms) following a limb perturbation during a voluntary upper limb movement. Leinonen et al. (2002) recorded responses of the T_12_-L_1_ level *M. erector spinae* (~50 ms) and the L_5_-S_1_ level *M. multifidus* (~35 ms) that occurred simultaneously or even before the movement of the same trunk level (T_12_, ~47 ms).
